# Hirudin Alleviates Early Brain Injury After Subarachnoid Hemorrhage in Rats via Regulating NLRP3 Inflammasome-Mediated Pyroptosis

**DOI:** 10.33549/physiolres.935454

**Published:** 2025-04-01

**Authors:** Mingfu PAN, Haiping CHEN, Yang ZHAI, Wei LONG, Yuandai LUO

**Affiliations:** 1 Zhuang Medicine Classic Ward, Guangxi International Zhuang Medicine Hospital, Nanning, China

**Keywords:** Subarachnoid hemorrhage, Early brain injury, Hirudin, pyroptosis, Nod-like receptor protein 3 (NLRP3) inflammasome

## Abstract

Subarachnoid hemorrhage (SAH) is a critical neurological emergency and one of the leading causes of stroke. Neuronal demise serves as the primary factor contributing to early brain injury (EBI) following SAH. This study aims to investigate the molecular mechanism underlying Hirudin’s impact on EBI after SAH, with a particular focus on pyroptosis. The SAH rat model was established by performing intravascular puncture, followed by the administration of Hirudin and Nod-like receptor protein 3 (NLRP3) agonist Nigericin into the lateral ventricle. The SAH grading, neurological score, brain water content, blood-brain barrier (BBB) permeability, neuronal damage, inflammatory reaction, neuronal death, distribution of microglia marker Iba-1 and expression levels of NLRP3 inflammasomal-related proteins were evaluated at 72 h post-SAH. Hirudin treatment significantly ameliorated neurological scores and attenuated brain edema, BBB permeability, inflammatory response, microglia activation, and pyroptosis in SAH rats. Additionally, Hirudin treatment downregulated the expression levels of NLRP3 inflammasomal-related proteins, such as NLRP3, apoptosis-associated speck-like protein (ASC) and cleaved caspsase-1. However, Nigericin partially counteracted the aforementioned effects of Hirudin, indicating that Hirudin exerted its inhibitory effect on pyroptosis by modulating the NLRP3 inflammasome pathway. The neuroprotective effect of Hirudin on EBI following SAH is attributed its ability to inhibit pyroptosis mediated by NLRP3 inflammasome, suggesting its potential as a promising therapeutic approach for SAH.

## Introduction

Subarachnoid hemorrhage (SAH) is a critical neurological emergency, and it is additionally recognized as one of the prevalent etiologies contributing to stroke [1]. The formation of intracranial aneurysms occurs through the dilation of intracranial arteries, which are susceptible to rupture due to diminished elasticity in the blood vessel wall. Consequently, aneurysm rupture serves as a significant precipitating factor for SAH [2]. The mortality and disability rates associated with SAH are significant [3]. Despite advancements in neurological and endovascular surgery technology and management, the impact of SAH on patients’ lives and health remains substantial, imposing a considerable burden on individuals and society [4]. More than 25 % of patients succumb to mortality during the early stage of SAH [5]. The pathological progression of SAH can be primarily categorized into two stages: early brain injury (EBI) and delayed cerebral ischemia [6]. Research has demonstrated that EBI following SAH is closely associated with secondary complications and unfavorable prognosis; nevertheless, there lacks a specific treatment approach [7]. Consequently, therapeutic investigations targeting EBI hold immense significance in enhancing outcomes for SAH.

Pathological processes observed during EBI encompass blood-brain barrier disruption, microcirculatory dysfunction, neuroinflammatory responses, cerebral edema formation, and neuronal demise [8]. A plethora of studies have demonstrated that the regulation of neuroinflammation holds promising prospects for the treatment of brain injury following SAH [9–11]. Pyroptosis is a pro-inflammatory programmed cell death, which was once considered to be caspase-dependent apoptosis. However, in 2001, it was discovered that pyroptosis and apoptosis exhibit distinct characteristics: apoptotic cells maintain an intact plasma membrane due to cell shrinkage, whereas pyroptotic cells experience swelling leading to rupture of the plasma membrane [12,13]. The hallmark features of pyroptosis cells include cellular swelling, formation of cell membrane pores, membrane rupture, and subsequent release of cellular contents [14]. This process is accompanied by the release of proinflammatory cytokines, such as interleukin-1β (IL-1β) and interleukin-18 (IL-18), which not only amplify the inflammatory effects but also activate the immune response [15,16]. Neuronal pyroptosis plays a crucial role in the pathogenesis and progression of central nervous system-related disorders, and represents one of the key mechanisms underlying brain injury [17–20]. Activation of the Nod-like receptor protein 3 (NLRP3) inflammasome can mediate neuronal pyroptosis and contribute to the progression of brain injury [21,22]. The clinical data demonstrates an excessive activation of the NLRP3 inflammasome in patients with SAH [23]. Furthermore, other studies have indicated that inhibiting the activation of the NLRP3 inflammasome can significantly enhance brain injury recovery and reduce pyroptosis in nerve cells following SAH [24,25]. Consequently, targeting the NLRP3 inflammasome may prove to be a promising therapeutic approach for treating SAH.

The small protein Hirudin, consisting of 64–66 amino acids, is a highly active and extensively researched constituent found in leeches and their salivary glands [26]. It exhibits a broad spectrum of pharmacological effects including anticoagulant and antithrombotic properties, enhancement of microcirculation, promotion of metabolism, regulation of blood lipid metabolism, reduction in blood glucose levels, as well as improvement in myocardial ischemia and arrhythmia. Currently, research has demonstrated the therapeutic efficacy of Hirudin in cerebral infarction, cerebral hemorrhage, and cerebral ischemia-reperfusion [27–29]. Its mechanism of action encompasses the inhibition of neuroinflammation, reduction of oxidative stress levels, promotion of hippocampal nerve regeneration, and facilitation of hematoma absorption. Nevertheless, it remains uncertain whether Hirudin exerts a beneficial effect on SAH. Numerous studies have demonstrated that Hirudin exerts inhibitory effects on the activation of NLRP3 inflammasome [27,30,31]. Therefore, we postulate that Hirudin may ameliorate brain injury following SAH by suppressing NLRP3 inflammasome-mediated pyroptosis.

In this study, we established an experimental rat model of SAH to investigate the impact of Hirudin on EBI in these rats and explore the underlying mechanism by which Hirudin affects pyroptosis in brain neurons of SAH rats, aiming to provide novel insights for the development of EBI treatment strategies following SAH.

## Materials and methods

### Animals

The adult Sprague-Dawley rats (male, 8 weeks old, weighing 200–220 g) were procured from Hubei Laboratory Animal Research Center. The rats were housed under standard conditions: temperature maintained at 21 ± 1 °C, humidity at 55 ± 5 %, with a 12-h light/dark cycle and ad libitum access to food and water. Model establishment commenced after one week of acclimatization.

### SAH model and experiment design

The study involved a total of 80 rats, which were divided into five groups: Sham group (*n*=12), SAH group (*n*=17), SAH + Hirudin group (*n*=17), SAH + Nigericin group (*n*=17), and SAH + Hirudin + Nigericin group (*n*=17). The rats in all groups except for the Sham group were subjected to experimental construction of the SAH model.

The SAH model was established in rats by puncturing the internal carotid artery, based on previous studies [32]. Briefly, the rats were weighed and anesthetized with intraperitoneal injection of 2 % sodium pentobarbital (40 mg/kg) (Sigma-Aldrich Corp., USA). Following fixation, a 2.0 cm longitudinal incision was made along the midline of the right anterior cervical triangle to separate the cervical vessels (common carotid artery, external carotid artery, and internal carotid artery) in sequence. The external carotid artery was ligated and transected, followed by the insertion of a suture from the vascular side of the stump into the internal carotid artery via the bifurcation of the common carotid artery. The suture encountered resistance after being advanced approximately 2.0 cm into the vessel, and was further advanced by an additional 3 mm, resulting in puncturing of the vessel wall of the internal carotid artery. After a duration of 10 s, the suture line was withdrawn, and sequential ligation and suturing were performed on the lateral incision of the external carotid artery. Rats in the Sham group were only subjected to vascular separation without puncture.

The rats in the SAH+Hirudin group received Hirudin (Sigma-Aldrich) injections of 10 U into the lateral ventricle at 30 min, 24 h, and 48 h post-SAH. The Hirudin dosage was determined based on prior research study [33]. The injection coordinates for the lateral ventricle were as follows: 1.0 mm posterior to the bregma, 1.4 mm lateral to the midline, and 4.4 mm below the surface of the skull [34]. The drug was administered into the ventricle at a rate of 1 μl/min, and the needle was left in place for 5 min following each injection to prevent reflux. The rats in the SAH+ Nigericin group were injected with Nigericin (2 μg/rat, MedChemExpress, USA) into the lateral ventricle at 30 min post-SAH. The Nigericin dosage was determined based on prior research study [35]. The rats in the SAH+Hirudin+Nigericin group were given Hirudin combined with Nigericin. The rats in the Sham group and SAH group received intracerebroventricular injections of an equal volume of phosphate buffer solution (PBS).

### SAH grade

The severity of SAH in rats was evaluated and graded at 72 h post-surgery, following the grading criteria established in previous study [32]. Briefly, rats were anesthetized with sodium pentobarbital and euthanized, after which their brains were promptly extracted. The basal cisterna was divided into six sections and assigned scores ranging from 0 to 3 based on the presence of subarachnoid clots within each section. Finally, the scores from all six sections were summed up. Grade 0 indicated no blood clot present in the subarachnoid space; Grade 1 represented a small amount of blood clot; Grade 2 denoted a moderate clot with identifiable arteries; Grade 3 signified complete coverage of all arteries within the segment by the clot.

### Brain edema measurement

After the completion of the aforementioned assessment, the right cerebral hemisphere tissues of rats were carefully placed onto an electronic analytical balance in order to accurately determine wet weight. Subsequently, the brain tissues were subjected to a drying process in an oven at 105 °C for 24 h and weighed again to obtain their dry weight. Brain water content was then calculated as (wet weight − dry weight)/(wet weight) × 100 %.

### Behavioral assessment

The modified Garcia score [36] and balance beam test were employed to assess the neurological function of rats. The modified Garcia score was divided into six components: spontaneous activity, symmetry in the movement of four limbs, forepaw outstretching, body proprioception, response to vibrissae touch, and climbing test. The first three components were assessed on a scale of 0 to 3, while the remaining three components were evaluated on a scale of 1 to 3. Finally, the total score for all six components was calculated. For the balance beam test, rats were placed on a 1 m high and 12 mm wide beam, and their performance was assessed based on the distance covered in 1 min, using a scoring system ranging from 0 to 4. Three consecutive trials were conducted, and the average score was calculated.

### Evans blue extravasation

The blood-brain barrier (BBB) permeability in rats was evaluated using the Evans blue extravasation method as previously described [37]. In brief, rats were anesthetized with intraperitoneal injection of 2 % sodium pentobarbital (40 mg/kg) at 72 h after SAH. Subsequently, a 2 % Evans blue solution (2 ml/kg, MedChemExpress) was administered via the left femoral vein. After circulating for 60 min, the rats were sacrificed and their brain tissues were promptly isolated. The tissues were homogenized using saline and then centrifuged at 12,000 g for 25 min. The resulting supernatant was collected and mixed with an equal volume of trichloroacetic acid before being incubated overnight at 4 °C. Following centrifugation at 12,000 g for 25 min, the supernatant was collected and Evans blue dye was quantified by photometry at a wavelength of 610 nm.

### Hematoxylin and eosin (HE) staining

Rat brain tissues were removed from 4 % paraformaldehyde (Solarbio, Beijing), embedded in paraffin, and sectioned (4 μm thick). The selected sections from cerebral cortical regions were mounted on slides and subjected to HE staining (Solarbio) for the purpose of observing brain injury.

### Immunofluorescence staining

The cerebral cortex sections were deparaffinized, treated with Triton X-100 (Beyotime, Shanghai), and blocked by incubating in 3 % bovine serum albumin (BSA, Beyotime) for 30 min. The sections were then subjected to immunostaining using anti-Iba-1 monoclonal antibody (diluted at 1:100, Abcam, UK) overnight at 4 °C. Afterward, the secondary antibody was applied and incubated for 50 min at room temperature in the dark. 4′,6-diamidino-2-phenylindole (DAPI) staining solution (Solarbio) was added and incubated for 10 min at room temperature in the dark. Finally, the expression of Iba-1 in the brain tissues was observed under a fluorescence microscope (OLYMPUS, Japan). The percentage of Iba-1 positive cells was quantified using Image J software.

### Terminal-deoxynucleotidyl transferase mediated nick end labeling (TUNEL) staining

After deparaffinizing the sections of cerebral cortex, proteinase K (Beyotime) was added and incubated at 37 °C for 22 min. Triton X-100 was introduced to disrupt the membrane, followed by BSA blocking. The TUNEL assay solution (Beyotime) was then applied and incubated in the dark at 37 °C for 1 h. Nuclei were stained with DAPI staining solution and incubated at room temperature in the dark for 10 min. Finally, cell death in brain tissue was observed under a fluorescence microscope. The percentage of TUNEL positive cells was quantified using Image J software.

### Enzyme linked immunosorbent assay (ELISA)

The cryopreserved cerebral cortex tissues were harvested, homogenized, and centrifuged at 12,000g for 5 min to collect the supernatants. Subsequently, the levels of tumor necrosis factorα (TNF-α), Interleukin-6 (IL-6), Interferon-γ (INF-γ), IL-1β, and IL-18 in the tissues were determined using ELISA kits (Elabscience, Wuhan) following the provided instructions.

### Western blot analysis

Cryopreserved cerebral cortex tissues were collected and total proteins were extracted using RIPA lysate containing protease inhibitors (Beyotime). After quantification, 20 μg of protein was utilized as the loading volume for sodium dodecyl sulfate-polyacrylamide gel electrophoresis (SDS-PAGE). The protein was then transferred onto a polyvinylidene difluoride membrane and blocked with 5 % skimmed milk for 1 h at room temperature. After washing the membranes, they were incubated overnight at 4 °C with primary antibodies against NLRP3 (1:1,000, Abcam), apoptosis-associated speck-like protein (ASC) (1:1,000, Abcam), Caspase1 (1:200, Santa Cruz Biotechnology, USA) and GAPDH (1:5,000, Abcam), followed by a 1h incubation with secondary antibodies at room temperature. Chemiluminescence reagent was added to develop the protein bands, and Image J software was utilized to analyze the gray value of the protein bands.

### Statistical analysis

All data were analyzed using SPSS 21.0 software and expressed as mean ± standard deviation (SD). One-way analysis of variance (ANOVA) followed by the LSD-test were used to evaluated the significant differences among multiple groups. *P*<0.05 was considered statistically significant.

## Results

### The Mortality and SAH grade

**Fig. 1:**
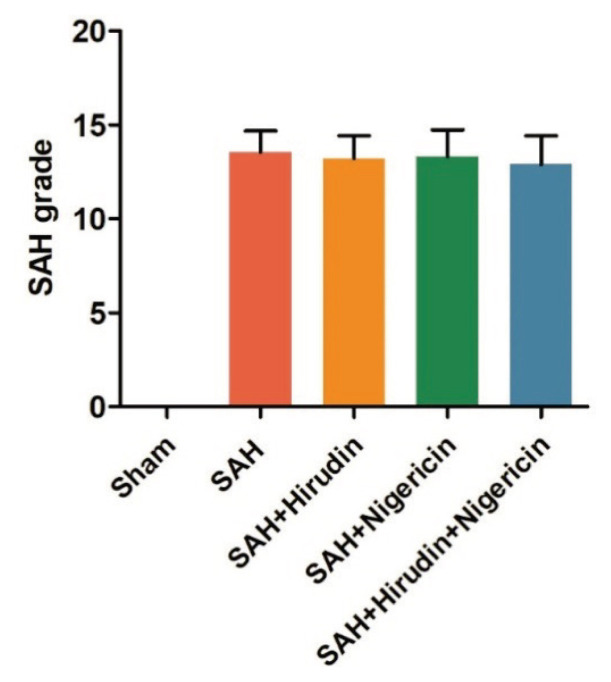
Statistical analysis of SAH grade in rats. After the SAH model was established, Hirudin and/or Nigericin were administered via intracerebroventricular injection in rats, and brain tissues were collected 72 h later for SAH grading (*n*=9).

At the conclusion of modeling and drug administration in this study, no mortality was observed in the Sham group, while 4 out of 17 rats died in the SAH + Nigericin group (4/17), along with 3 deaths in both the SAH + Hirudin group and the SAH group (3/17). Additionally, 4 rats died in the SAH + Hirudin + Nigericin group (4/17). In addition, the SAH grade did not differ significantly between the other groups, except for the sham group (Fig. 1).

### Hirudin ameliorated brain injury in rats with SAH

**Fig. 2:**
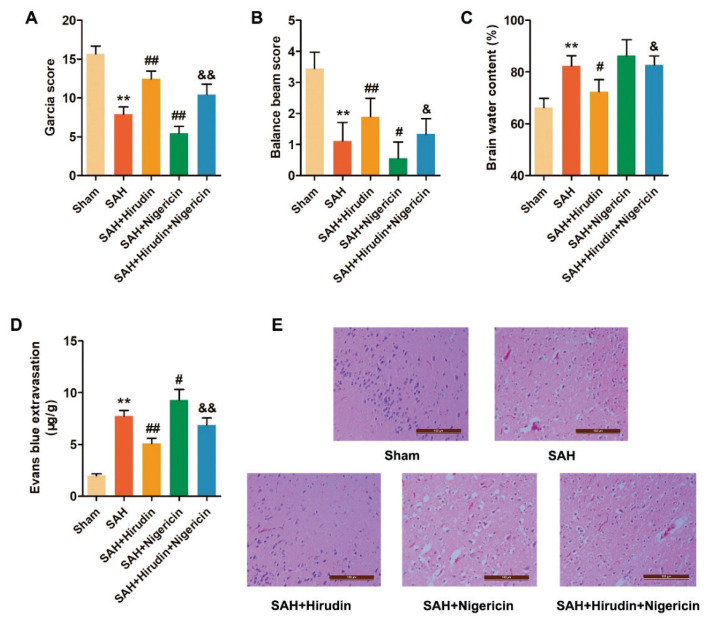
Hirudin improved EBI in SAH rats. At 72 h after SAH, the effects of Hirudin on (**A**) Garcia score (*n*=9), (**B**) balance beam score (*n*=9), (**C**) brain water content (*n*=3) and (**D**) Evans blue extravasation (*n*=3) were evaluated in rats. (**E**) HE staining was used to observe the effect of Hirudin on pathological damage of cerebral cortex in rats with SAH (*n*=3). Scale bar=100 μm. ^**^P<0.01 vs. Sham group. ^#^*P*<0.05, ^##^*P*<0.01 vs. SAH group. ^&^*P*<0.05, ^&&^*P*<0.01 vs. SAH+Hirudin group.

In order to investigate the impact of Hirudin on brain injury in SAH rats, we assessed behavioral experiments, brain edema, BBB permeability, and pathological observations. The data from the behavioral experiments revealed that both the Garcia score and balance beam score of SAH rats were significantly lower compared to those of the Sham group. Following Hirudin treatment, there was a decrease in both the Garcia score and balance beam score of SAH rats; however, Nigericin treatment increased these scores and partially reversed the effect of Hirudin (Fig. 2A,B). Similarly, Hirudin treatment alleviated cerebral edema and reduced Evans blue extravasation in experimental SAH rats. Conversely, Nigericin treatment exacerbated cerebral edema and Evans blue extravasation in SAH rats, while also partially counteracting the effects of Hirudin (Fig. 2C,D).

Additionally, HE staining revealed disorganized arrangement and abnormal morphology of neurons in the cerebral cortex of SAH rats, characterized by condensed nuclei, disappearance of nucleoli, cellular edema, and extensive infiltration of inflammatory cells. Hirudin mitigated the pathological damage to the cerebral cortex in SAH rats, whereas Nigericin exerted an opposing effect and partially reversed the impact of Hirudin (Fig. 2E). In summary, Hirudin demonstrated a mitigating effect on the EBI induced by SAH in rats, whereas the NLRP3 agonist Nigericin exacerbated the brain injury and partially counteracted the protective impact of Hirudin on SAH-induced brain injury.

### Hirudin inhibited microglia activation and inflammatory response in cerebral cortex of rats with SAH

**Fig. 3:**
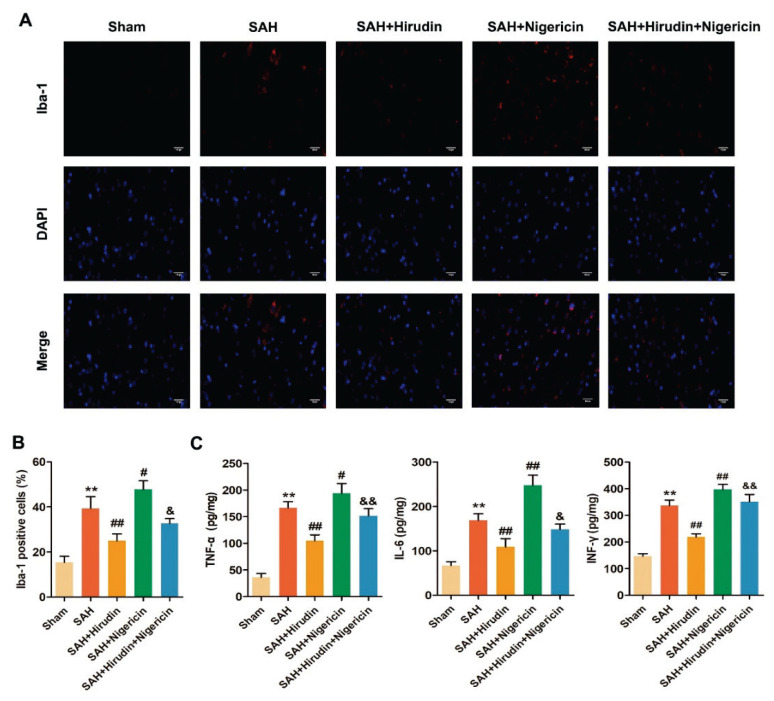
Hirudin inhibited neuroinflammation in SAH rats. (**A–B**) At 72 h after SAH, the expression of Iba-1 in the cerebral cortex was observed using immunofluorescence staining to evaluate the activation level of microglia (*n*=3). Scale bar=100 μm. Red fluorescence indicates Iba-1 positive expression, blue fluorescence indicates nucleus. (**C**) The levels of TNF-α, IL-6, and INF-γ in the cerebral cortex were assessed using ELISA (*n*=3). ^**^P<0.01 vs. Sham group. ^#^*P*<0.05, ^##^*P*<0.01 vs. SAH group. ^&^*P*<0.05, ^&&^*P*<0.01 vs. SAH+Hirudin group.

Activated microglia may serve as the instigator of neuroinflammation [38]. Hence, immunofluorescence staining was employed to examine the expression of Iba-1, a marker for microglia, in the brain tissue of rats with SAH. The findings revealed an increase in Iba-1 positive cells within the cerebral cortex of the SAH group, as compared to the Sham group (Fig.3A, 3B). Notably, Hirudin treatment decreased the quantity of Iba-1 positive cells, while Nigericin augmented their presence, Furthermore, co-treatment with Nigericin reversed the effect of Hirudin. We further assessed the levels of proinflammatory cytokines TNF-α, IL-6 and INF-γ using ELISA. The results revealed a significant elevation in TNF-α, IL-6 and INF-γ levels within the cerebral cortex tissue of SAH rats compared to the Sham group. However, Hirudin treatment effectively attenuated these cytokines levels. Conversely, Nigericin treatment exacerbated TNF-α, IL-6 and INF-γ levels in the cerebral cortex of SAH rats while partially reversing the inhibitory effects of Hirudin on neuroinflammation in this model (Fig. 3C). In short, Hirudin exhibited suppressive effects on neuroinflammation in SAH rats, whereas the NLRP3 agonist Nigericin promoted neuroinflammation in SAH rats and partially counteracted the effects of Hirudin.

### Hirudin suppressed pyroptosis mediated by NLRP3 inflammasome activation in rats with SAH

**Fig. 4:**
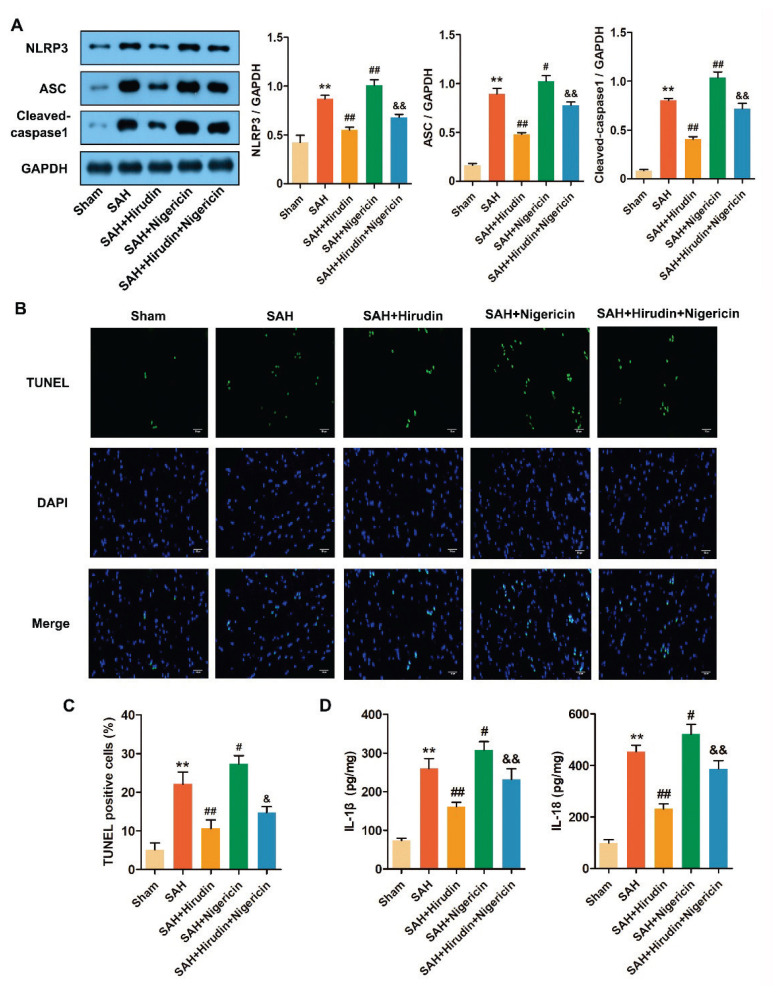
Hirudin inhibited neuronal pyroptosis mediated by NLRP3 inflammasome activation in SAH rats. (**A**) At 72 h after SAH, the protein expression levels of NLRP3, ASC, and Cleaved-caspase1 in the cerebral cortex were assessed using Western blot analysis, and statistical analysis was performed (*n*=3). (**B–C**) Neuronal pyroptosis in the cerebral cortex was observed using TUNEL staining (*n*=3). Scale bar=100 μm. Green fluorescence indicates pyroptotic cells, blue fluorescence indicates nucleus. (**D**) The levels of IL-1β and IL-18 in the cerebral cortex were assessed using ELISA (*n*=3). ^**^P<0.01 vs. Sham group. ^#^*P*<0.05, ^##^*P*<0.01 vs. SAH group. ^&^*P*<0.05, ^&&^*P*<0.01 vs. SAH + Hirudin group.

Since NLRP3 agonist Nigericin partially reversed the effects of hirudin on EBI after SAH in the above experimental results, we hypothesized that the mechanism of action of hirudin on EBI might be related to the regulation of NLRPS inflammasome-mediated pyroptosis. Western blot analysis confirmed this hypothesis, revealing significantly elevated protein expression levels of NLRP3, ASC, and Cleaved-caspase1 in the cerebral cortex of SAH rats compared to the Sham group. Treatment with Hirudin effectively inhibited NLRP3 inflammasome activation; however, administration of Nigericin further stimulated NLRP3 inflammasome activity and partially counteracted the inhibitory effects of Hirudin (Fig. 4A). Additionally, TUNEL staining revealed an augmented occurrence of cell death in the cerebral cortex of SAH rats, which was mitigated by Hirudin treatment but exacerbated by Nigericin administration. Furthermore, Nigericin partially counteracted the inhibitory effect of Hirudin on cell death (Fig. 4B,C). ELISA results demonstrated that Hirudin treatment significantly suppressed the levels of IL-1β and IL-18 in the cerebral cortex of SAH rats, whereas Nigericin exerted an opposite effect (Fig.4D). The findings suggest that Hirudin exerts an inhibitory effect on pyroptosis mediated by the activation of NLRP3 inflammasome in rats with SAH, which can be reversed by the NLRP3 agonist Nigericin.

## Discussion

This study represents the first investigation into the neuroprotective effect of Hirudin and its correlation with NLRP3 inflammasome-mediated pyroptosis in an animal model of SAH. The findings demonstrate that SAH effectively protects EBI and suppresses microglia activation-induced neuroinflammation in rats with SAH. Additionally, SAH also inhibits NLRP3 inflammasome activation, thereby mitigating pyroptosis.

EBI refers to the development of total brain damage within 72 h following SAH, and it represents one of the primary pathological factors contributing to mortality and unfavorable prognosis. The BBB serves as a crucial protective interface between the blood microcirculation system and the brain parenchyma, safeguarding neurons. Inflammatory mediators play a pivotal role in modulating BBB permeability [39]. The presence of neuroinflammation enhances blood-brain barrier permeability, thereby resulting in subsequent cerebral edema [40]. The disruption of the BBB, cerebral edema, and neuroinflammation constitute the primary pathological processes underlying EBI [6]. The rat model of SAH was successfully established in this study. At 72 h post-SAH, the rats exhibited neurological dysfunction, increased BBB permeability, brain edema, and histological staining revealed inflammatory cell infiltration in the cerebral cortex. The use of Hirudin as a specific thrombin inhibitor in clinical studies has been well-established [41]. Extensive research on the pharmacological effects of Hirudin has revealed its therapeutic potential in brain tissue damage diseases when applied locally [27–29]. However, there is limited literature available regarding the application of hirudin in SAH. This study represents the first report demonstrating that Hirudin treatment can ameliorate pathological symptoms associated with EBI, although further investigation is required to elucidate the underlying mechanism.

The inflammatory response is a crucial process in the management of numerous threatening diseases, exhibiting its positive role in restoring tissue homeostasis and promoting tissue healing [42]. However, under pathological conditions, the prolonged persistence of the inflammatory response can lead to secondary tissue damage, thereby exacerbating the disease [43]. The role of neuroinflammation following SAH in the development and progression of EBI is widely acknowledged [44,45]. Microglia, as resident immune cells in the brain, aggregate and become activated immediately following SAH. The neuroinflammation mediated by microglia plays a crucial role in EBI subsequent to SAH [46]. It has been found that during the EBI stage, neuroinflammation invages the brain through activated microglia and spreads to all layers of the cerebral cortex [47]. We also observed a significant increase in the population of Iba-1 positive microglia in the cerebral cortex of SAH rats, accompanied by an upregulation in their secretion levels of pro-inflammatory cytokines TNF-α, IL-6, INF-γ, and IL-1β. Li *et al*. discovered that Hirudin demonstrated a regulatory effect on microglial NLRP3 inflammasome-mediated neuroinflammation in both *in vivo* and *in vitro* models of acute cerebral ischemia [27]. Given this finding, we postulated that Hirudin may exert inhibitory effects on neuroinflammation in SAH rats through the regulation of NLRP3 inflammasome activation, thereby ameliorating EBI.

The occurrence of neuronal death is regarded as a crucial initiator for the pathological progression of EBI [48]. The molecular mechanism underlying neuronal death in brain injury following SAH is intricate and encompasses multiple pathways of cell death, including pyroptosis [49]. The key distinction between pyroptosis and apoptosis lies in the disruption of plasma membrane integrity, leading to extracellular release of cellular contents, which is implicated in the pathogenesis of various human inflammatory disorders. Nevertheless, similar to apoptosis, pyroptosis exhibits DNA fragmentation, enabling visualization of pyroptotic cells through TUNEL assay [50]. A growing body of research has demonstrated a correlation between neuronal pyroptosis and the excessive activation of NLRP3 inflammasome in animal models of SAH [51,52]. The NLRP3 inflammasome is composed of NLRP3, Caspase1, and ASC. Upon sensing danger signal stimulation, NLRP3 undergoes oligomerization and binds to ASC at its NH_2_-terminus, subsequently recruiting pro-caspase1 to form the inflammasome complex [53,54]. The formation of the inflammasome leads to autocatalysis and activation of pro-caspase1. Cleaved-caspase1, which is enzymatically active, not only processes the precursors of cytokines IL-1β and IL-18 into their mature secreted forms but also cleaves the N-terminus of Gasdermin D to create a pore in the cell membrane. This subsequently results in the release of intracellular IL-1β and IL-18 outside the cell, leading to pyroptosis [55,56]. The potential of Hirudin in regulating NLRP3 inflammasoma-mediated pyroptosis is significant [30,31], yet its regulatory effect on NLRP3 inflammasomas at the EBI after SAH remains unclear. In our experiments, Hirudin treatment demonstrated a reduction in pyroptosis and a decrease in protein expression of NLRP3, ASC, Cleaved-caspase1, as well as IL-1β and IL-18 formation in SAH rats. Interestingly, the inhibitory effect of Hirudin on pyroptosis mediated by NLRP3 inflammasome activation was partially reversed by the NLRP3 agonist Nigericin. In addition, the neuroprotective effect of Hirudin treatment was found to be impeded in the presence of Nigericin.

In conclusions, our findings suggest that hirudin treatment effectively improves EBI in rats with SAH, partly by inhibiting the activation of NLRP3 inflammasome and subsequently reducing pyroptosis. However, certain limitations still exist. Firstly, our findings do not provide conclusive evidence for the regulatory role of Hirudin in NLRP3 inflammasome activation in microglia. Secondly, our study specifically focused on investigating the neuroprotective effect of Hirudin on SAH through the lens of NLRP3 inflammasome activation, thereby excluding exploration into other pathways associated with Hirudin.
